# Delayed fractional dose regimen of the RTS,S/AS01 malaria vaccine candidate enhances an IgG4 response that inhibits serum opsonophagocytosis

**DOI:** 10.1038/s41598-017-08526-5

**Published:** 2017-08-11

**Authors:** Sidhartha Chaudhury, Jason A. Regules, Christian A. Darko, Sheetij Dutta, Anders Wallqvist, Norman C. Waters, Erik Jongert, Franck Lemiale, Elke S. Bergmann-Leitner

**Affiliations:** 1 Biotechnology High Performance Computing Software Applications Institute, Telemedicine and Advanced Technology Research Center, US Army Medical Research and Materiel Command, Fort Detrick, MD USA; 2 Department of Clinical Research, US Army Medical Research Institute of Infectious Diseases, Ft. Detrick, MD USA; 3 Malaria Vaccine Branch, US Military Malaria Research Program, Walter Reed Army Institute of Research, Silver Spring, MD USA; 4 GSK Vaccine, Rixensart, Belgium; 5 PATH Malaria Vaccine Initiative, Washington DC, USA

## Abstract

A recent study of the RTS,S malaria vaccine, which is based on the circumsporozoite protein (CSP), demonstrated an increase in efficacy from 50–60% to 80% when using a delayed fractional dose regimen, in which the standard 0–1–2 month immunization schedule was modified to a 0–1–7 month schedule and the third immunization was delivered at 20% of the full dose. Given the role that antibodies can play in RTS,S-induced protection, we sought to determine how the modified regimen alters IgG subclasses and serum opsonophagocytic activity (OPA). Previously, we showed that lower CSP-mediated OPA was associated with protection in an RTS,S study. Here we report that the delayed fractional dose regimen resulted in decreased CSP-mediated OPA and an enhanced CSP-specific IgG4 response. Linear regression modeling predicted that CSP-specific IgG1 promote OPA, and that CSP-specific IgG4 interferes with OPA, which we subsequently confirmed by IgG subclass depletion. Although the role of IgG4 antibodies and OPA in protection is still unclear, our findings, combined with previous results that the delayed fractional dose increases CSP-specific antibody avidity and somatic hypermutation frequency in CSP-specific B cells, demonstrate how changes in vaccine regimen alone can significantly alter the quality of antibody responses to improve vaccine efficacy.

## Introduction

The core desired attribute of a malaria vaccine is the ability to reliably induce long-lasting and sterile immunity against infection. Targeting the pre-erythrocytic sporozoite stage of *Plasmodium* is thought to combat infection between the site of the mosquito bite and the liver, thereby preventing the onset of morbidity and stopping the parasite’s transmission cycle. Immunity induced by a pre-erythrocytic vaccine is sterile and, thus, drastically differs from natural immunity whereby parasites persist in the blood of infected individuals without invoking severe symptoms of the disease.

The circumsporozoite protein (CSP) is one of the main targets of anti-sporozoite antibodies when immunity is induced by whole-sporozoite vaccine candidates, such as sporozoite immunization under chloroquine cover^1^ or radiation-attenuated sporozoites^2^. CSP is the most abundantly expressed protein on the surface of the sporozoite. It has been the leading malaria vaccine antigen for decades, albeit with variable success depending on the vaccine platform^3–5^. RTS,S/AS01, which is the current lead recombinant vaccine candidate against malaria, is based on a pseudo-particle consisting of the hepatitis B surface antigen and the central repeat and C-terminal regions of CSP. The vaccine consistently induces 50–60% sterile protection in malaria-naïve individuals^6^; however, for it to become a viable commercial product, higher efficacy levels are needed in light of the continuing morbidity and mortality caused by malaria. Moreover, a highly efficacious anti-infection vaccine would play an important role in accelerating parasite eradication.

To date, there are no confirmed immune correlates of protection against malaria infection. The identification of immune correlates would greatly assist in vaccine design and down-selection of vaccine candidates. There is, however, mounting evidence that antibodies to the repeat region in RTS,S are associated with protection against malaria^6^, although it is unknown how these antibodies may contribute to protection. Previous studies have shown that while CSP-specific antibodies may be a surrogate marker for vaccine efficacy^6^, CSP-specific antibody titers alone are insufficient for predicting protection^3, 7, 8^, suggesting that while CSP-specific antibodies are essential for vaccine efficacy, some qualitative aspects of RTS,S-induced antibody responses, such as their fine specificity, avidity, isotype, and functional activity, play a major role in determining protection.

Our laboratory is developing functional assays to evaluate humoral and cellular responses induced by vaccination in an effort to reveal immune correlates of protection. We previously reported on the inverse association between serum opsonophagocytic activity (OPA) mediated by RTS,S-induced antibodies and protection against sporozoite challenge, employing a sensitive, high-throughput *in vitro* OPA assay^7^. The study analyzed OPA in serum samples from an RTS,S trial, in which roughly 50% of the study participants were protected from a controlled human malaria infection (CHMI). These experiments guided the development of the OPA index used here, which is expressed as a log ratio of the OPA titer and the ELISA titer. We found that the OPA index was significantly lower in protected individuals. The data encouraged us to apply this readout tool to additional RTS,S studies in an effort to validate the OPA index as a surrogate marker of protection.

A recent clinical trial^8^ showed that a delayed fractional dose regimen of RTS,S, in which the standard 0–1–2 month immunization schedule was altered to a 0–1–7 schedule, with the third immunization at 20% of the full dose. This change in the standard immunization regimen results in a significant increase in vaccine efficacy, reproducing a result first observed two decades ago^9^. The clinical trial revealed that modifying the vaccine regimen alone, without altering the vaccine formulation, can have a dramatic effect on protective immunity: compared to the standard regimen (012M), the delayed fractional dose regimen (Fx017M) demonstrated a significant increase in vaccine efficacy from 63% (95% CI, 29.4–80.1%) to 86.7% (95% CI, 66.8–94.6%). Furthermore, the study showed that the Fx017M regimen resulted in an increase in CSP-specific antibody avidity and higher somatic hypermutation frequency in CSP-specific B cells^8^. Here, we used serum samples from this trial to expand on our previous findings that the OPA index may be a potential surrogate marker of protection^7^.

The present study was designed to profile the serological response induced by the different regimens through the assessment of the functional activity of CSP-specific antibodies and by integrating the results with other serological measures, such as titer, avidity, and IgG subclasses, to determine the mechanistic basis for the dramatic increase in vaccine efficacy. Although we were unable to confirm the OPA index as a surrogate marker of protection, in part because of the low number of non-protected individuals in this study, we identified two additional factors that differentiate the Fx017M cohort from the 012M cohort: 1) induction of IgG4 antibodies and 2) significantly lower OPA. Importantly, the finding that CSP-specific IgG4 titers were predominantly detected in the Fx017M cohort prompted additional mechanistic experiments to determine the interplay between the IgG subclasses and their impact on biological activity. Using linear regression modeling, we showed that CSP-specific IgG1 and IgG3 antibodies were positively associated with OPA, whereas IgG4 antibodies were negatively associated with OPA. We subsequently carried out IgG subclass depletion experiments and found that depletion of IgG4 antibodies resulted in significantly higher OPA, particularly in sera from the Fx017M cohort. Our results suggest that CSP-specific IgG4 can directly reduce the OPA of other IgG subclasses. The study further explores the utility of OPA as a surrogate marker for efficacy in CSP-based malaria vaccines and its value in identifying potentially new mechanisms of protection.

## Results

### Serum OPA

We employed a previously developed *in vitro* assay to measure the serum OPA mediated by immune sera from subjects immunized with the RTS,S vaccine^7^. Briefly, CSP-coated fluorescent beads were incubated with the serum sample at five different dilutions (1:200, 1:400, 1:800, 1:1600, 1:3200) for 1 hr, washed, and then added to THP-1 cells, a human monocytic cell line which expresses receptors comparable to competent macrophages^10^. During the 1 hr incubation with the THP-1 cells, opsonized CSP-coated beads were phagocytosed. OPA was then quantified by flow cytometry through two related measures: macrophage frequency (Mfreq), which measures the percentage of the THP-1 population that phagocytosed one or more CSP-coated beads; and mean fluorescent intensity (MFI), which measures the average number of fluorescent beads taken up by phagocytosing THP-1 cells (see Methods). Using the OPA at each serum dilution, we calculated the MFI and Mfreq OPA titer, as the serum dilution that corresponds to 60% of peak opsonization activity.

**Figure 1:**
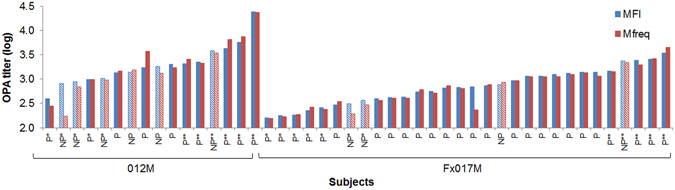
Phagocytic activity by subjects in 012M and Fx017M cohorts. OPA, as measured by MFI (red) and Mfreq (blue) OPA titers for each subject in the 012M and Fx017M cohorts, denoted by their protection status as protected (‘P’, solid bars) or not protected (‘NP’, hatched bars). Subjects selected for low and high phagocytic activity pools are shown as ‘*’ and ‘**’, respectively, for both cohorts.

We tested serum samples from subjects in the 012M (n = 16) and Fx017M (n = 30) cohorts of a recent RTS,S clinical trial^8^. The MFI and Mfreq OPA titers of each subject are shown in Fig. 1, along with the protection status. As reported previously^7^, we found a strong correlation between MFI and Mfreq (R = 0.85), suggesting that both measures share similar underlying mechanisms.

**Figure 2:**
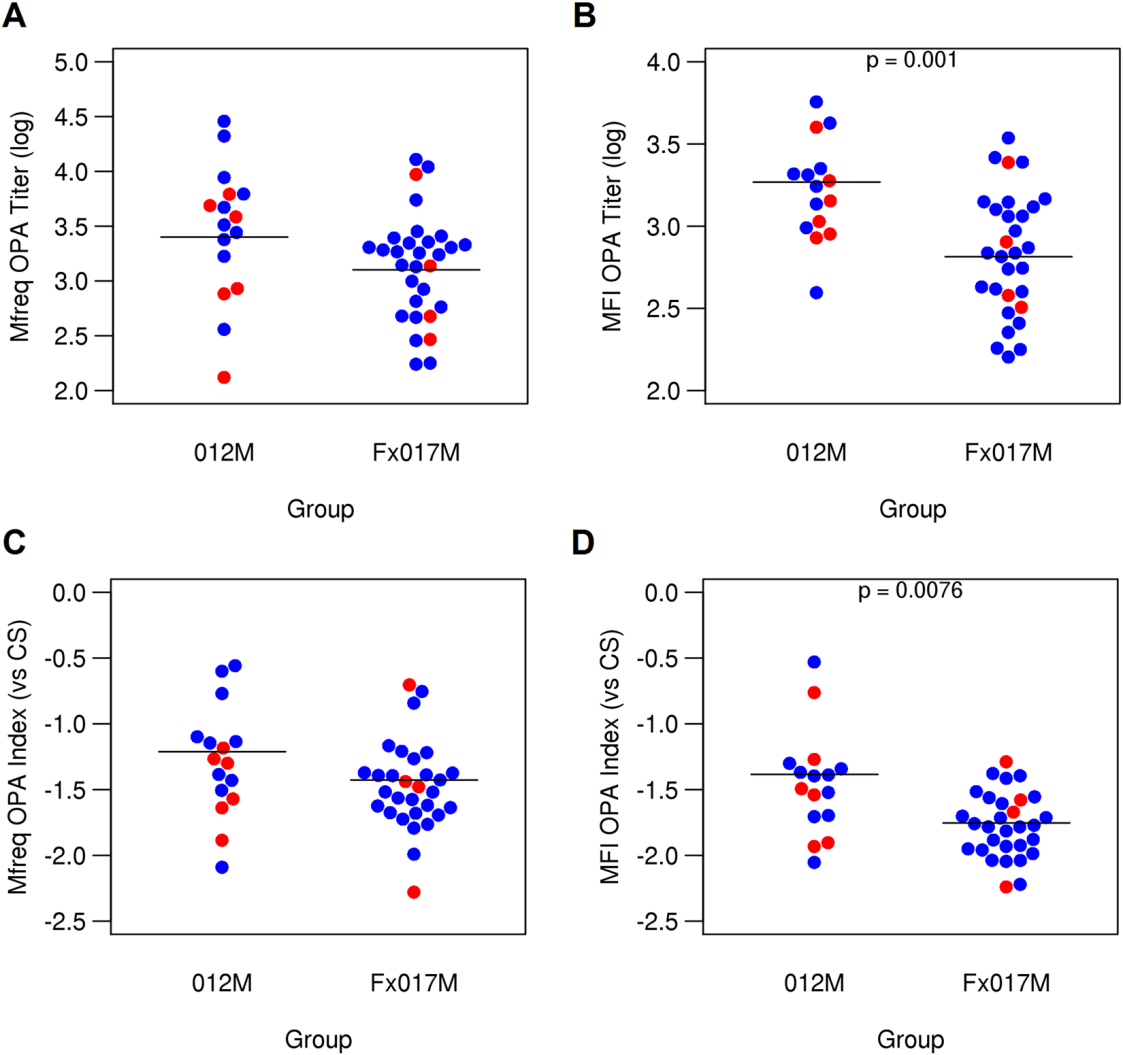
Opsonophagocytic activity in 012M and Fx017M cohorts. OPA titers for Mfreq (**A**) and MFI (**B**) for the 012M and Fx017M cohorts. OPA indices for Mfreq (**C**) and MFI (**D**), relative to CS antibody titer, for 012M and Fx017M cohorts. Protected and non-protected subjects are shown in blue and red, respectively.

Overall, the Fx017M cohort showed significantly lower OPA titers than the 012M cohort, using both MFI and Mfreq as measures (p < 0.05, and p < 0.01, respectively) (Fig. 2). We previously reported that a relative measure of OPA, known as the OPA index, was significantly lower in protected individuals in another RTS,S vaccine study. The OPA index expresses the functional activity of sera as the OPA titer relative to the CSP-specific antibody titer^7^, which is determined by ELISA. In the present study, the MFI OPA index was significantly lower in the 017M cohort than in the 012M cohort. The Mfreq OPA index tended to be lower in the Fx017M cohort, although the difference was not statistically significant.

In a previous RTS,S study, we reported an association between OPA and C-terminal antibodies^7^, based on the observation that OPA correlated with full-length CSP (FL-CSP) and C-terminal region ELISA titers, but not with repeat region ELISA titers. In this study, we could not reproduce this aspect; instead, OPA correlated with ELISA titers from all three antigens in both cohorts, and unlike in the previous study, the correlation *between* each ELISA measure was also higher (data not shown). It is unclear, however, whether this was due to differences in the ELISA assay conditions between the two studies.

### Serum IgG isotype subclass MFIs

**Figure 3:**
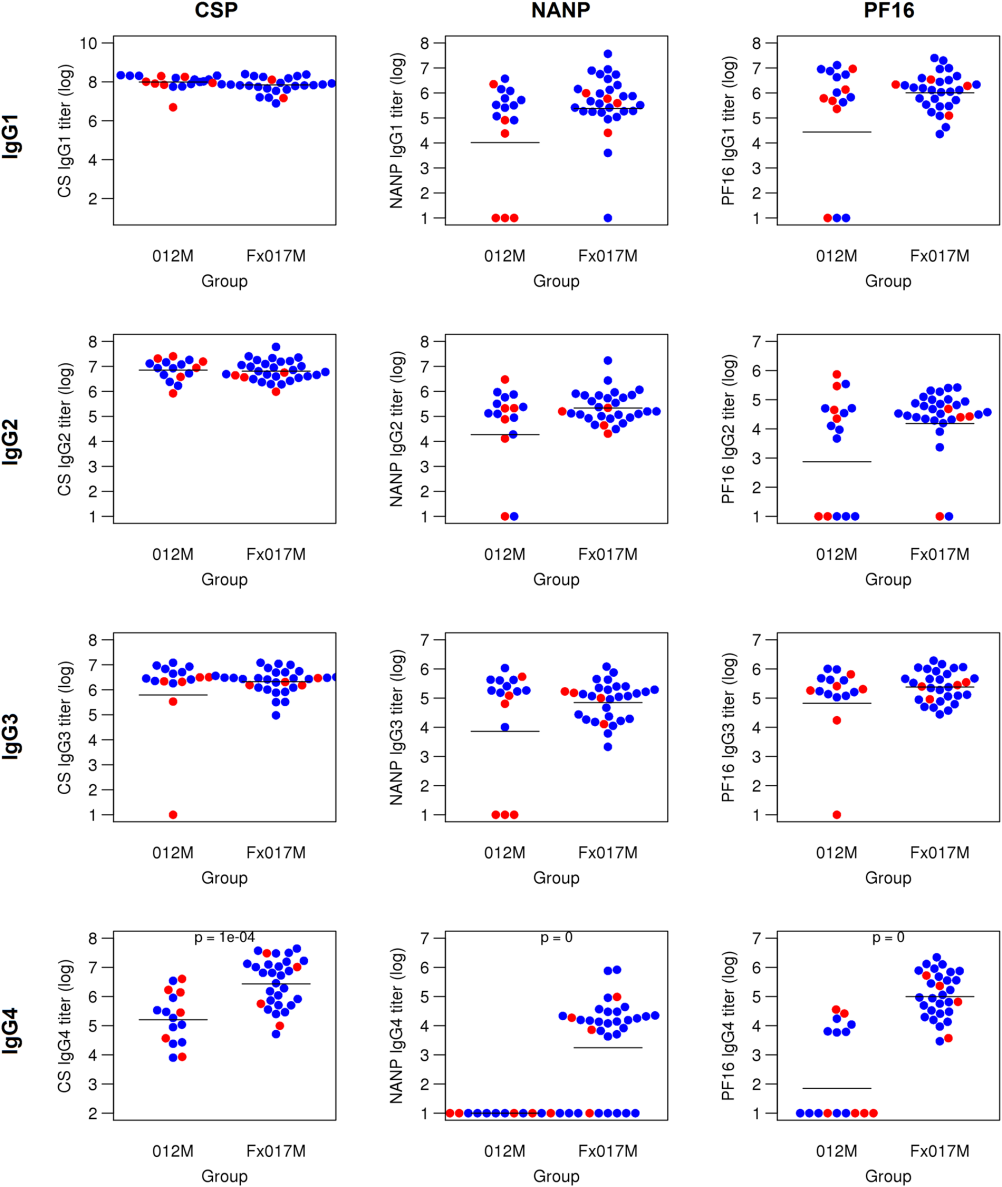
IgG subclass titers for 012M and Fx017M cohorts. Antibody titers for IgG1, IgG2, IgG3, and IgG4 for the 012M and Fx017M cohorts against three CSP test antigens: full-length recombinant CSP (left column), NANP repeat region peptide (middle column), and C-terminal PF16 peptide (right column). Protected and non-protected subjects are colored in blue and red, respectively.

We measured the IgG subclass MFIs for each serum sample, using a Luminex-based assay against three CSP test antigens: full-length recombinant CSP, a peptide representing the repeat region (NANP), and a peptide representing the C-terminal region (PF16) (Fig. 3). Overall, there was no significant difference in IgG1, IgG2, or IgG3 titers between the 012M and Fx017M cohorts, for all three CSP antigens. However, the Fx017M cohort showed significantly higher IgG4 MFIs than the 012M cohort (p < 10^−4^) when sera were tested against the full-length CSP test antigen. This effect was also seen with the NANP and PF16 antigens: no subject in the 012M cohort showed detectable IgG4 responses to NANP, whereas a majority of subjects from the Fx017M cohort (14 out of 20) showed robust IgG4 MFIs to NANP. For the C-terminal PF16 antigen, only 50% of subjects in the 012M cohort showed detectable IgG4 titers, whereas all subjects in the Fx017M cohort did. In conclusion, the Fx017M cohort differed serologically from the 012M cohort, with its significantly enhanced IgG4 response against all three CSP test antigens.

### Linear regression model

Antibody function is determined by the Fc region of the various immunoglobulin isotypes. The structure this region, as well as its glycosylation pattern, governs which Fc receptors are engaged by the immunoglobulin/immune complex. We sought to identify the contribution of the CSP-specific IgG subclasses to OPA. Initially, we used a computational approach (linear regression modeling) to address this question (Table 1). Because our assay to measure OPA is based on fluorescent beads coated with full-length recombinant CSP (FL-CSP) to determine functional activity, we focused our analysis on antibody titers against FL-CSP. For linear regression modeling, we implemented two models. In Model 1, the dependent variable, OPA titer (either Mfreq or MFI), was modeled as a linear combination of the following independent variables: IgG1, IgG2, IgG3, and IgG4 titers on the data set of all subjects in both cohorts combined. The model assigned a weight to each IgG isotype subclass titer that reflects the relative contribution of that variable, as well as a p-value reflecting the statistical significance of its contribution. In Model 2, we replaced IgG4 titer with an independent ‘dummy’ variable to represent the cohort identity (‘group dummy’) of 012M (group dummy = 0) or Fx017M (group dummy = 1).

**Table 1:** Linear regression model for factors contributing to OPA.

Dependent variable:	OPA titer (Mfreq)		OPA titer (MFI)	
Independent variable	Model 1	Model 2	Model 1	Model 2
IgG1	1.05***	1.02***	0.76***	0.67***
IgG2	0.10	0.07	0.01	–0.09
IgG3	0.01	0.03	0.03	0.07
IgG4	–0.10*		–0.15*	
Group dummy		−0.11*		−0.32**
*Summary Statistics*				
No. of observations	46	46	46	46
R^2^	0.59	0.59	0.55	0.62

^*^p < 0.05; **p < 0.01; ***p < 0.001.

Linear regression modeling using Model 1 revealed that for both Mfreq and MFI, IgG1 showed a strong positive association with OPA titer (p < 10^–2^), whereas IgG4 was negatively associated with OPA (p < 0.05). With Model 2, we found that the Fx017M cohort was negatively associated with OPA. The model results show that there is a modest, negative, relationship between OPA and IgG4, once the contribution of IgG1, IgG2, and IgG3 titers is accounted for (Supplementary Figure S1), across all individuals in both cohorts. From these results, we hypothesized that IgG1 was primarily responsible for OPA whereas IgG4 inhibits opsonization, and that the lower OPA of the Fx017M cohort was due to inhibition by IgG4 antibodies.

### IgG isotype subclass depletion experiments

The linear regression model identified correlations or associations that served as the basis for our hypothesis on the contributions of the different IgG subclasses to OPA. To experimentally validate our hypothesis, we carried out IgG isotype subclass depletion experiments, whereby IgG1, IgG3, or IgG4 antibodies were selectively depleted from a serum sample prior to testing for OPA. Because of limitations in the amount of serum available from each subject and the need for a relatively large quantity of serum for these experiments, we needed to pool serum samples for the depletion experiments. One risk with using an aggregate readout from a pooled sample is that the contribution of any single individual sample to the pooled sample may be masked by other samples in the pool. For example, a serum sample with low OPA might be masked by a serum sample with high OPA if they were combined into a single pooled sample and assessed for OPA. For this reason, we created pooled subsets of low- and high-OPA subjects into a low-OPA pool (low-OPA) and a high-OPA pool (high-OPA), respectively, for the 012M and Fx017M cohorts (see Fig. 1), which resulted in four test samples. This way, we could assess the role of IgG subclass depletion with respect to both overall serum OPA (low vs. high) and cohort (012M and Fx017M), independently.

We carried out the depletion experiments two times, and present both the average results as well as the lower and higher values for each measure from the two independent runs. We found that the depletion experiment resulted in high reproducibility for Mfreq OPA measures between the two runs (R^2^ = 0.85), but produced poor reproducibility for the MFI OPA measure (R^2^ = 0.23), particularly for the low OPA pools (R^2^ = 0.05). We have previously reported that Mfreq OPA measure shows more reliability than the MFI OPA measure^7^, but the reasons for the particularly poor reproducibility of the MFI measure of OPA following depletion of the serum sample are unclear. Therefore, we restricted our analysis of the effects of IgG subclass depletion to Mfreq measures of OPA.

**Figure 4:**
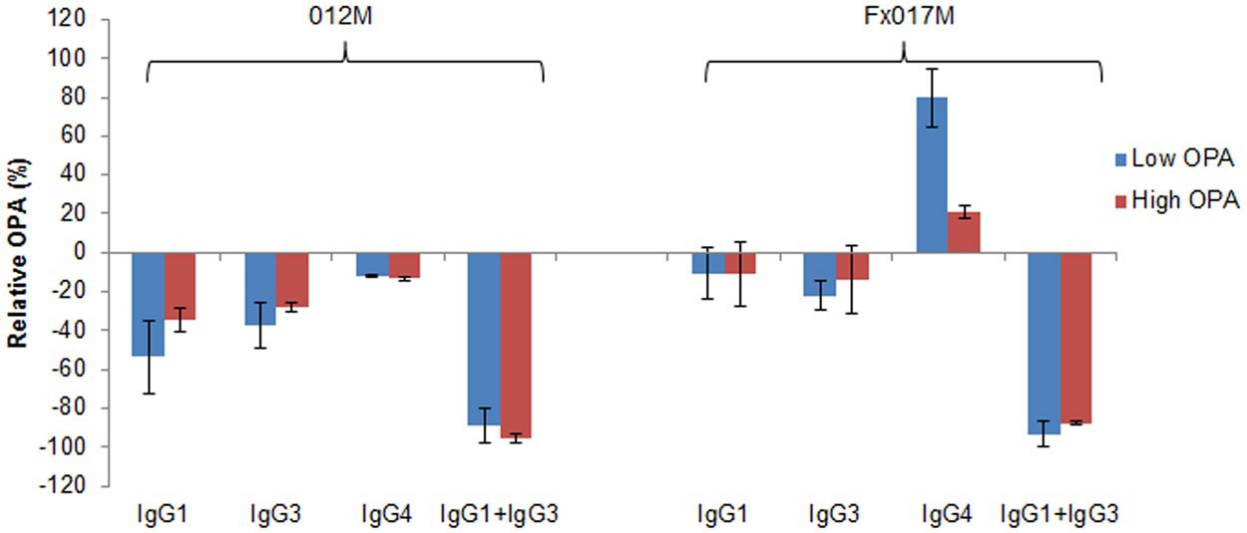
Serum opsonophagocytic activity following depletion of IgG isotype subclass antibodies. The change in peak OPA relative to the non-depleted sample, as measured by Mfreq, when the low-OPA and high-OPA pools from the 012M and Fx017M cohorts were depleted of IgG1, IgG3, IgG4, or IgG1 + IgG3.

We measured the relative change in OPA in terms of Mfreq, relative to the same sample without depletion, at a serum dilution of 1:250 (Fig. 4). Depletion of either IgG1 or IgG3 alone led to a 0 to 50% reduction in OPA, whereas the combined IgG1 + IgG3 depletion led to a reduction of 80 to 100%, suggesting that at a serum dilution of 1:250, IgG1 and IgG3 antibodies act in a redundant manner to contribute to OPA. Furthermore, IgG4 depletion led to an increase in OPA in the Fx017M cohort, particularly for the low-PA pool (+80%), suggesting that in this cohort, IgG4 antibodies may be strongly inhibiting OPA and, thus, are associated with protection in the Fx017M regimen. These results confirm the linear regression modeling that IgG1 contributes to OPA and that IgG4 inhibits OPA, but also shows that IgG3 plays a major role in OPA, a finding not predicted by the modeling. Subsequent analysis of the modeling results showed that IgG1 and IgG3 titers are correlated (R^2^ = 0.25), and that combining the two measures resulted in a significant positive association with OPA titers (*p* < 0.001) using both Model 1 and Model 2.

**Figure 5:**
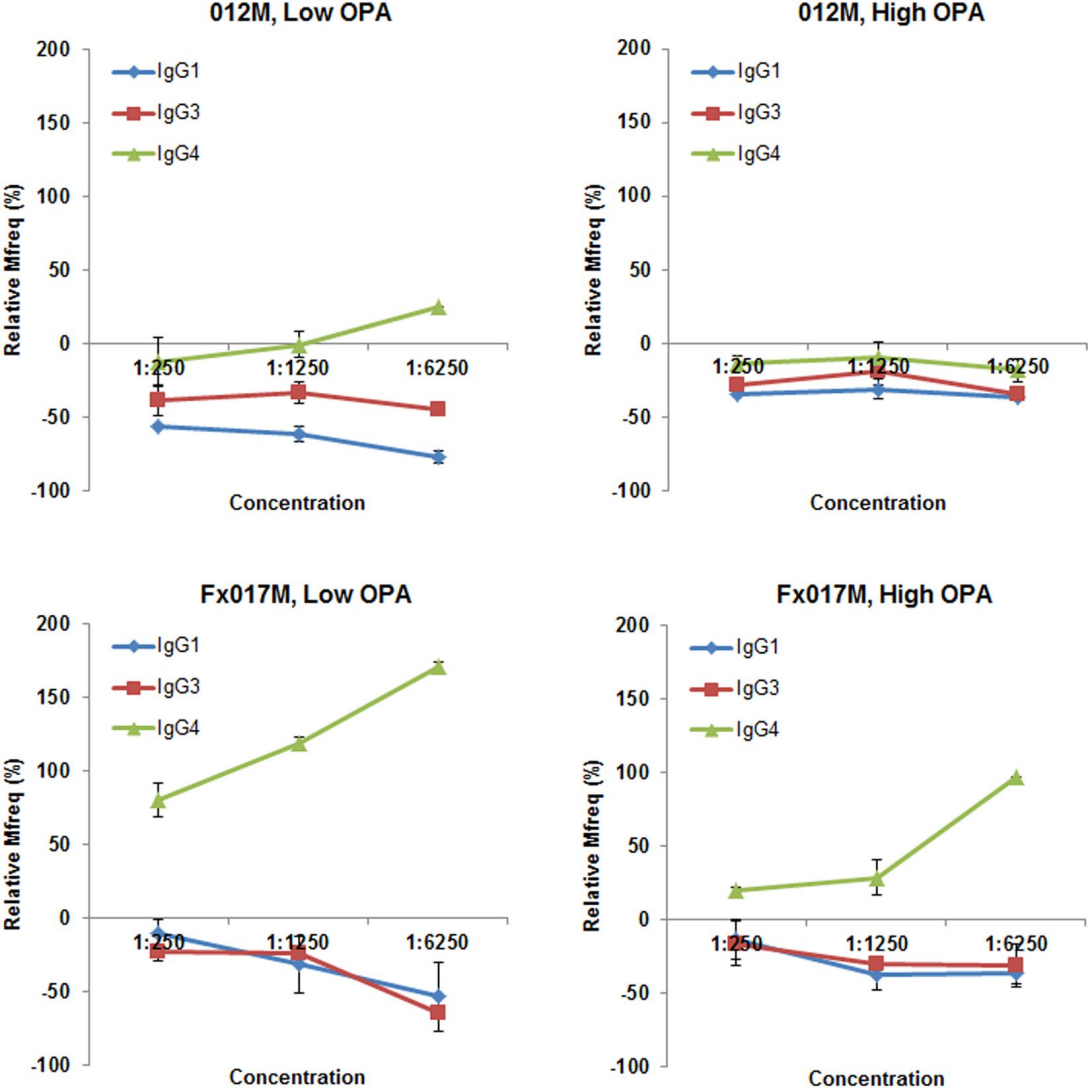
Serum opsonophagocytic activity following depletion of IgG isotype subclasses at a range of serum concentrations. The change in Mfreq relative to the non-depleted sample is shown following depletion of IgG1, IgG3, and IgG4 for the low-OPA and high-OPA pools for the 012M and Fx017M cohorts.

We measured the change in serum OPA following IgG isotype subclass depletion at several concentrations (1:250, 1:1250, 1:6250). Figure 5 shows the change in Mfreq relative to the non-depleted sample for depletion of IgG1, IgG3, and IgG4 within the range of serum concentrations tested. As the serum concentration decreased, the enhancement of OPA following IgG4 depletion increased, particularly in the Fx017M cohort. In the low-OPA Fx017M pool, IgG4 depletion led to an increase in OPA from +70% at 1:250 dilution to +185% at 1:6250 dilution. In the high-OPA Fx017M pool, IgG4 depletion increased OPA from +15% at 1:250 dilution to +100% at 1:6250 dilution. Finally, the low-OPA pool showed a greater increase in OPA following IgG4 depletion than did the high-OPA pool for both the 012M and Fx017M cohorts. Depletion of IgG1 and IgG3 antibodies led to a 20% to 70% reduction in OPA in all cohorts. The reason for this concentration-dependent effect is unclear; it is possible that at lower serum concentrations, the higher-avidity IgG4 outcompetes the lower-avidity cytophilic IgG1 and IgG3 for antigen binding, or that IgG1/IgG3-mediated OPA is more sensitive to changes in concentration than is IgG4-mediated inhibition, for example, if OPA requires the formation of a multivalent Fc-receptor engagement whereas inhibition does not. In conclusion, IgG4 antibodies substantially inhibit OPA, especially in the Fx017M cohort.

## Discussion

The identification of immunological markers that reliably predict protective, sterile immunity against many infectious diseases has been a major challenge^11^. Knowing what immune measures are predictive of protection would greatly accelerate vaccine development and reduce the number of clinical trials necessary to identify protective vaccine formulations. In a previous study, we found that low serum OPA in individuals vaccinated with RTS,S was associated with protection^7^, based on the analysis of serum samples from a clinical trial with 20 subjects immunized with the standard regimen of RTS,S/AS01B (0, 1, 2 months; 012M) given at full dose^12^.

In the present study, we pursued several objectives. First, we sought to determine whether the candidate surrogate marker of OPA applied broadly to CSP-based malaria vaccines and would still apply when the immunization regimen was changed. Second, we designed the study to better understand the mechanism underlying the significant increase in protection against malaria associated with a change in the vaccination regimen. We obtained samples from a recently published clinical trial that had two arms: a standard regimen (012M) and a delayed fractional dose regimen (Fx017M). The subsequent CHMI demonstrated that vaccine efficacy was significantly improved in the delayed fractional dose regimen, which prompted us to use samples from this clinical trial for serological profiling.

We profiled the humoral responses of 36 study participants by (1) quantitatively assessing antibody titers against FL-CSP, the repeat region, and the C-terminus^8^; (2) measuring the avidity of CSP-specific antibodies^8^; (3) determining CSP-specific IgG1, IgG2, IgG3, and IgG4 MFIs; and (4) quantifying serum OPA. Using linear regression modeling, we identified serological factors that significantly contributed to serum OPA in the study. We identified key differences between the two vaccine regimen cohorts and hypothesized that the enhanced IgG4 responses in the Fx017M cohort was responsible for the reduced OPA compared to the 012M cohort, which we subsequently confirmed using IgG isotype depletion experiments. This is of particular interested in light of our finding in a previous study that low OPA was a surrogate marker of protection in CSP-based vaccines^7^ (which was also reported by Boyer *et al*. unpublished observation).

The clinical study and the associated analyses highlight the impact of vaccine dose and schedule on the type and quality of the induced immune response and on protective efficacy. The immunological assessment of the recent clinical trial demonstrated that the Fx017M cohort had a significantly higher rate of somatic hypermutation in the RTS,S-vaccine-induced plasma cells as well as higher CSP-specific antibody avidities^8^. Further clinical studies are needed to determine whether the fractional booster dose alone, or the extension of the interval between the second and third immunizations, or both are responsible for the increase in vaccine efficacy and the underlying immunological changes.

Several theories may explain why the delayed fractional dose results in increased IgG4 responses and decreased OPA, and how this may be linked to protection. The reduced antigen load in the third delayed dose may have induced resting memory B cells expressing high-avidity IgG to start differentiating into high-avidity IgG4-secreting B cells. This assumption contrasts with the prevalent view that induction of IgG4 is favored by extended or chronic exposure to antigen in a non-infectious setting^13^. It has been postulated that the typically high-affinity IgG4 subclass outcompetes other subclasses and thereby interferes with any Fc-receptor mediated activations/events^14^. Unlike the switch to other antibody isotypes, the induction of IgG4 cannot be explained simply by the cytokines produced by T helper subsets. Instead, other factors such as the prolonged presence of antigen^14^ or inflammation as well as the presence of IL-12 during a TH2 response favor the induction of IgG4^15^. Extending the immunization regimen may represent the prolonged exposure of the immune system to the CSP antigen. This theory is consistent with our model-based prediction and subsequent experimental confirmation that IgG1 and IgG3 antibodies were responsible for antibody OPA, whereas IgG4 antibodies inhibited IgG1- and IgG3-mediated OPA. Finally, it is important to note that a protective role of IgG4 against infection has been previously reported for a potentially therapeutic monoclonal antibody capable of blocking sporozoite invasion of hepatocytes^16^.

Currently, information on the role of antibody subclasses in mediating immunity against pre-erythrocytic stages of *Plasmodium* is scarce. However, numerous studies have described the anti-parasitic effects of antibodies against erythrocytic stages, indicating that IgG4 antibodies may either block the invasion of host cells or inhibit the intracellular development of the parasites through various mechanisms^17, 18^. Similarly, the binding of CSP-specific antibodies to sporozoites has been shown to inhibit their motility^19^. In a study utilizing *in vivo* imaging in mice, we previously demonstrated that CSP-specific antibodies, induced by DNA immunization, block invasion of the liver^20^. It remains to be determined whether other vaccine platforms that elicit CSP-based immunity induce similar immune mechanisms. RTS,S-mediated immunity has been associated with antibodies as well as CD4^+^ T cells^6^, consistent with our rationale in profiling the humoral immune response.

While the hypothesis that opsonized sporozoites are targeted for phagocytosis is plausible as a mechanism of protection, data from our previous study showed a modest, but statistically significant, *inverse* relationship between OPA and protection^7^, indicating that any possible role that OPA plays in protection is more complex. The current study was underpowered in terms of the sample size needed to reproduce the previously-observed relationship between OPA and protection based on the effect size observed in the previous study. However, we did find that OPA was significantly lower in the sera from subjects in the Fx017M cohort, which had significantly higher efficacy than the 012M cohort. This regimen induced significantly higher efficacy than the 012M regimen, which is consistent with a link between lower OPA and protection. It is possible that phagocytosis mediated by CSP-specific antibodies may not contribute to protection, but instead constitute an immune escape mechanism, similar to that shown in another apicomplexan, *i.e*., *Leishmania* ^21^, in which parasite-specific proteins convert the complement factor C3b and thereby promote enhanced binding to complement receptor (CR) 3. The preferential binding to CR3 rather than to CR1 prevents the oxidative burst response during phagocytosis and ultimately results in the escape of the parasite^22, 23^. Finally, it is important to note that the *in vitro* assay used to assess OPA in this study has certain differences from possible *in vivo* serum OPA during sporozoite infection, including the differences in antigen presentation between the CSP-coated fluorescent beads and sporozoites, differences in phagocytic activity between THP-1 macrophages and host macrophages, and the role of other immune components not captured by the *in vitro* assay, such as complement or T cells.

Studies investigating the role of the CSP antigen in sporozoite infections of host cells demonstrate that the protein downregulates the immune function of liver-resident Kupffer cells^24, 25^, which are involved in the infection of the liver by sporozoites^26, 27^. The role of antibodies in the interaction of Kupffer cells and sporozoites, which leads to the uptake and safe passage sporozoites into the liver, has not yet been investigated. It is possible that CSP-specific IgG4 antibodies, which may have high avidity for CSP, outcompete cytophilic IgG1 and IgG3, preventing IgG1- and IgG3-mediated phagocytosis of the sporozoite. This, in turn, could prevent the sheltering of the sporozoite in phagocytic cells and its subsequent immune evasion or regulation of activity that interferes with protection.

## Materials and Methods

### Study summary

Serum samples were obtained from the clinical study NCT01857869. The efficacy and basic immunological evaluation of the trial have previously been reported^8^. Experiments were reviewed and approved by the GSK-MVI Correlates of Protection Task Force. All study participants had previously provided consent for future use of the samples for research. Consent to publish was not required because the samples were de-identified.

### ELISA assay for CS fine specificity

The ELISA assay was performed in the Malaria Serology Laboratory (USMMRP, WRAIR Silver Spring, USA), employing full-length CSP, NANP peptide, and C-terminal peptide (PF16) as plate antigens as previously described^28, 29^. ELISA titers are listed as endpoint dilutions at an optical density (OD) of 1.

### Immunoglobulin subclass analysis

Carboxylated xMAP Microspheres (Luminex Corporation, Austin, TX, USA) were coupled to malaria antigens, using the carbodiimide coupling technique. Serum samples were diluted appropriately and incubated with the coupled antigens. Human IgG1, IgG2, IgG3, and IgG4 antibody isotype subclasses were detected by separately adding R-phycoerythrin (PE)-conjugated mouse anti-human IgG1, IgG2, IgG3, or IgG4 IgG subclasses (Southern Biotech, Inc., Birmingham, AL, USA). Mean fluorescent intensity (MFI) was measured on a Luminex 200 equipped with xPonent 3.1 software (Luminex Corporation).

### Serum opsonophagocytic activity

Serum OPA was assessed by measuring the uptake of CSP-coated fluorescent beads by THP-1 cells as previously described^7^. Briefly, NeutrAvidin-coated fluorescent beads (1 µm size, excitation/emission = 488/530 nm, Molecular Probes, Eugene, OR, USA) were incubated with biotinylated CSP^30^ at 4 °C overnight. Beads were washed with PBS + 1% BSA and aliquots incubated with serially diluted sera (1:100, 1:500, 1:2,500, 1:12,500, 1:62,500) for 2 hrs at 4 °C, and then added to the THP-1 cells for 45 min (37 °C). Activity observed with the pre-immune serum (1:100 dilution) was used to determine the (non-specific) background activity for each study subject. Cells were analyzed on a FACSCalibur (CellQuest software, Becton Dickinson, Mountain View, CA, USA). The level of phagocytosis was determined by applying markers to the histograms. The marker M1 measures all fluorescent cells, whereas M2 reports cells that have taken up at least 2 beads. We reported the frequency of cells that underwent OPA (Mfreq), and the mean fluorescence intensity of cells that underwent OPA (MFI), which is related to the number of beads taken up, for M2 cells. We used Mfreq and MFI for M2 cells to ensure that the opsonization results reflected the most phagocytically active cells. However, overall we found a high correlation between Mfreq and MFI, as determined from M1 and M2 cells (R^2^ of 0.85 and 0.89 for Mfreq and MFI, respectively).

### IgG subclass depletion experiments

Four serum pools were generated: cohort 012M – low OPA (n = 5); cohort 012M – high OPA (n = 7); cohort Fx017M – low OPA (n = 5); and cohort Fx017M – high OPA (n = 5). NeutrAvidin-coated beads (non-fluorescent FluoSpheres® NeutrAvidin®-Labeled Microspheres, 1.0 μm, Molecular Probes) were incubated with biotinylated IgG subclass specific monoclonal antibodies (clone 8c/6–39 for IgG1, Sigma Aldrich, St. Louis, MO; CaptureSelect™ Biotin Anti-IgG3 for IgG3, Thermo Scientific, Waltham, MA; clone HP6025 for IgG4, Invitrogen, Rockford, IL). Beads were then used to deplete specific IgG subclasses from pooled sera in two subsequent incubations (overnight at 4 °C). Quality control for the depletion, *i.e*., measuring the remaining concentrations of the respective IgG subclasses (IgG1, IgG3, IgG4) after two subsequent rounds of depletion, was monitored using an Isotyping Kit for Immunoglobulins (Affymetrix, San Diego, CA) following the manufacturer’s instructions. Briefly, serial dilutions of the source material, and depleted fractions after the first and second rounds of absorption onto beads coated with capture antibodies, were tested for the presence of the respective IgG subclasses. The criterion for successful depletion was the failure of an ELISA signal at a 1:10 dilution of the material in the isotyping assay.

### Data analysis

We report the opsonization titer as the serum dilution that leads to 60% of peak opsonization activity, using either the Mfreq or MFI measure. At each dilution point, Mfreq and MFI were recorded in the M2 condition. For each OPA measure (Mfreq and MFI) and subject, we used a four-parameter logistic model^7^ to fit the opsonization activity (OPA) plotted as a function serum dilution (log[Ab]) and used the fitted curve to calculate the serum dilution at 60% maximal OPA. For each OPA measure, we used the OPA of the undiluted pre-immune sample for each subject as the background OPA, and subtracted it from the OPA at each dilution point, prior to calculating the OPA titers. We used the dilution at 60% maximal OPA because for a small number of subjects, the OPA at even the highest dilution was sufficiently high, such that a lower OPA (such as 50% of the maximum) would be outside the range of the data. Because there are two measures for OPA (Mfreq and MFI), we calculated two independent OPA titers for each subject.

### Statistics and modeling

Statistical significance was assessed using an unpaired Student’s t test, assuming unequal variances between protected and non-protected subjects. To describe correlations between measures, we reported the square of the Pearson correlation coefficient (R^2^) and the p-value following linear fitting. All analyses were carried out using the R statistical package. We used the lm function in R to perform linear regression modeling, with the opsonization activity measure (Mfreq or MFI) as the dependent variable; IgG1, IgG2, IgG3, and IgG4 titers as the independent variables; and Mfreq ~ (IgG1 + IgG3) + IgG2 + IgG4 and MFI ~ (IgG1 + IgG3) + IgG2 + IgG4 as the formulas connecting the dependent and independent variables. We also ran the linear regression by incorporating an independent dummy variable representing the vaccine arm, with values of 0 and 1 for the 012M and Fx017M cohorts, respectively.

### Data availability statement

The data used in this manuscript can be made available upon request.

### Ethical approval and informed consent

Serum samples were obtained from the clinical study NCT01857869 ﻿and the investigators have adhered to the policies for protection of human subjects as prescribed in AR 70-25. Experiments were reviewed and approved by the GSK-MVI Correlates of Protection Task Force. All study participants had previously provided consent for future use of the samples for research. Consent to publish was not required because the samples were de-identified.

## Electronic supplementary material

Supplementary Figure S1
