# Supplementary material for: Delayed fractional dose regimen of the RTS,S/AS01 malaria vaccine candidate enhances an IgG4 response that inhibits serum opsonophagocytosis
Source: Sci Rep. 2017 Aug 11;7:7998. doi: 10.1038/s41598-017-08526-5 (PMC5554171; doi:10.1038/s41598-017-08526-5)
Supplement: Supplementary file 1 — Supplementary Figure S1 [file 41598_2017_8526_MOESM1_ESM.doc]

# Supplementary Materials

# Delayed fractional dose regimen of the RTS,S/AS01 malaria vaccine candidate enhances an IgG4 response that inhibits serum opsonophagocytosis

Sidhartha Chaudhury^1^, Jason A. Regules^2^, Christian A. Darko^3^, Sheetij Dutta^3^, Anders Wallqvist^1^, Norman C. Waters^3^, Erik Jongert^4^, Franck Lemiale^5^, and Elke S. Bergmann-Leitner^3*^

Supplementary Figure S1


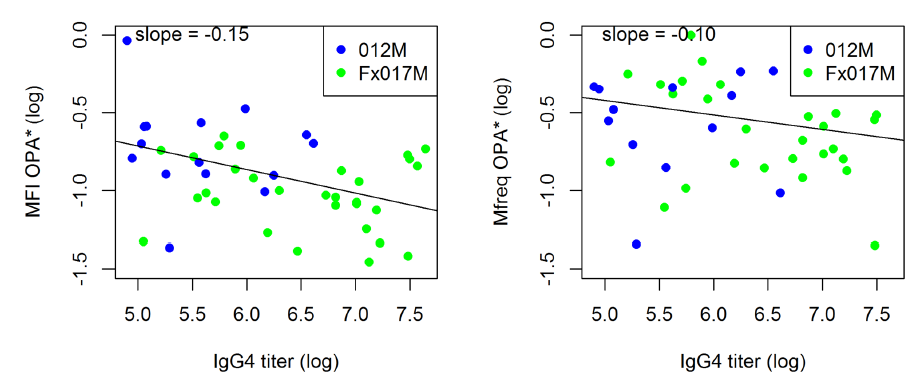
**Supplemental Figure S1. Negative relationship between IgG4 and OPA.** A scatterplot showing serum OPA after accounting for IgG1, IgG2, and IgG3 titers to MFI (MFI OPA*) and Mfreq (Mfreq OPA*) compared with IgG4 titer for all individuals in the 012M (blue) and Fx017M (green) cohorts. MFI OPA* and Mfreq OPA* were calculated by subtracting the modeled contribution of IgG1, IgG2, and IgG3 titers from OPA, for each individual, based on the linear regression model.”
